# Studies on the actin-binding protein HS1 in platelets

**DOI:** 10.1186/1471-2121-8-46

**Published:** 2007-11-09

**Authors:** Steven G Thomas, Simon DJ Calaminus, Jocelyn M Auger, Stephen P Watson, Laura M Machesky

**Affiliations:** 1 School of Biosciences, University of Birmingham, Edgbaston, Birmingham, B15 2TT, UK; 2 Centre for Cardiovascular Sciences, Institute of Biomedical Research, The Medical School, University of Birmingham, Edgbaston, Birmingham, UK

## Abstract

**Background:**

The platelet cytoskeleton mediates the dramatic change in platelet morphology that takes place upon activation and stabilizes thrombus formation. The Arp2/3 complex plays a vital role in these processes, providing the protrusive force for lamellipodia formation. The Arp2/3 complex is highly regulated by a number of actin-binding proteins including the haematopoietic-specific protein HS1 and its homologue cortactin. The present study investigates the role of HS1 in platelets using HS1^-/- ^mice.

**Results:**

The present results demonstrate that HS1 is not required for platelet activation, shape change or aggregation. Platelets from HS1^-/- ^mice spread normally on a variety of adhesion proteins and have normal F-actin and Arp2/3 complex distributions. Clot retraction, an actin-dependent process, is also normal in these mice. Platelet aggregation and secretion is indistinguishable between knock out and littermates and there is no increase in bleeding using the tail bleeding assay.

**Conclusion:**

This study concludes that HS1 does not play a major role in platelet function. It is possible that a role for HS1 is masked by the presence of cortactin.

## Background

The platelet is highly dependent upon its actin cytoskeleton for proper functioning. Dramatic re-arrangements of the actin cytoskeleton mediates spreading on matrix proteins and is required for normal thrombus formation [1,2]. At rest, the discoid shape of a platelet is maintained by a microtubule coil, a spectrin-based skeleton immediately below the plasma membrane, and a network of 2000 – 5000 actin filaments held rigid by the cross-linking proteins filamin and α-actinin [3-5]. Following Ca^2+ ^elevation, the actin-severing protein gelsolin is released from barbed ends leading to relaxing of the discoid shape and a large increase in the number of free barbed ends for polymerisation [6]. Concomitant activation of the Arp2/3 complex, a seven-membered protein complex which nucleates actin filaments, leads to a massive increase in the F-actin content of platelets. This provides the protrusive force for filopodia and lamellipodia formation that gives the platelet its characteristic spread morphology [7].

**Figure 1:**
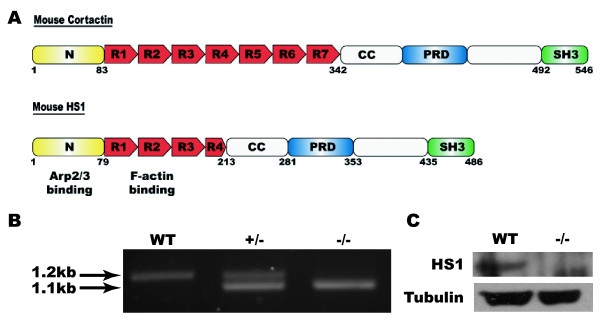
**Domain organisation of HS1^-/- ^and genotyping of knockout mice**. (A) Schematic representation of mouse cortactin and HS1 proteins. N – terminal acidic domain, R1, R2, etc – Cortactin repeats, CC – coiled coil helical domain, PRD – proline rich domain, SH3 – C-terminal Src homology domain. Numbers indicate amino acid number. (B) Genotyping of HS1 knockout mice by PCR. WT – wild type, HS1^+/- ^– heterozygote, HS1^-/- ^– homozygote. (C) Western blot of platelet extracts from WT and HS1^-/- ^mice probed with α-HS1 (top panel) and α-tubulin (bottom panel).

The Arp2/3 complex is regulated by a number of proteins which allow for tight spatial and temporal regulation of its activity, including haematopoietic lineage cell-specific protein 1 (HS1) and its homologue cortactin (for reviews see [8,9]) (Figure 1A). HS1 is expressed in cells of a haematopoietic lineage, whereas cortactin is ubiquitously expressed. Both proteins are regulated by tyrosine phosphorylation and have Arp2/3-binding, F-actin binding repeat, coiled coil, proline rich and C-terminal SH3 domains. However, cortactin has 6.5 F-actin binding repeats [8], whereas HS1 only has 3.5 and this changes the way in which the protein interacts with Arp2/3-induced F-actin arrays [10]. Similarly, the tyrosine residues which are phosphorylated are not conserved between the two proteins indicating that there are differences in their regulation [11,12].

HS1 is tyrosine phosphorylated downstream of T- and B-cell receptor activation [13] and following thrombin-stimulation of platelets [14]. Subsequent to phosphorylation in platelets, HS1 translocates to the plasma membrane [14] where it is postulated to be involved in the morphological changes observed during apoptosis [14,15]. In B- and T-cells, tyrosine phosphorylation is involved in the migration of HS1 to lipid rafts where it is proposed to mediate actin assembly [16]. HS1^-/- ^mice have normal lymphocyte development but are deficient in the proliferative response induced by immunoreceptor engagement. Gomez et al [17] have shown that in HS1^-/- ^T-cells the immune synapse, an F-actin and Arp2/3 containing structure [18], begins to form but is disorganised and does not persist. These studies indicate that HS1 may play a role in both signalling to actin assembly following signal perception and in maintenance of dendritic actin arrays downstream of Arp2/3 activation. In this study we utilised an HS1 gene knockout mouse (HS1^-/-^) to ask whether HS1 contributes to signalling by the platelet collagen receptor, GPVI, which signals through the same pathway as that used by immunoreceptors and also by other classes of platelet surface receptors.

## Results and discussion

### Genotyping

Wild type mice were identified by the production of a 1.2 kb PCR fragment using primers HS1-3'KO-S and HS1-KO-end-3' (Figure 1B). HS1^-/-^genotypes were detected by amplification of a 1.1 kb fragment resulting from insertion of the Lac-Z cassette into the gene [13] using primers HS1-3'KO-S and Lac-Z-3' (Figure 1B). Confirmation that HS1 protein was not expressed in these mice was obtained by western blotting using a rabbit polyclonal antibody raised against HS1 [17] (Figure 1C).

### Platelet spreading and actin organization

**Figure 2:**
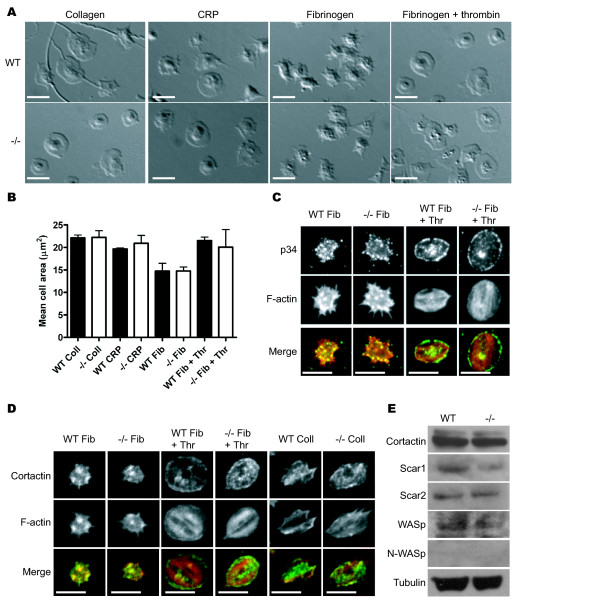
**Analysis of platelet spreading and F-actin organization**. Washed platelets (2 × 10^7 ^platelets/ml) from WT and HS1^-/- ^mice were added to cover slips coated with collagen (100 μg/ml), CRP (10 μg/ml) or fibrinogen (100 μg/ml) ± thrombin (1 U/ml) and allowed to settle for 45 or 90 min at 37°C. Spread platelets were fixed in formalin and imaged using DIC. Representative images of platelets at 45 min are shown in (A). Mean platelet area was measured. No significant difference was observed between WT and HS1^-/- ^platelets on any surface at 45 min (B). Platelets spread on fibrinogen ± thrombin for 45 min (C) were labeled with α-p34 for Arp2/3 complex (top panel), and rhodamine phalloidin for F-actin (middle panel). The merged images are shown in the bottom panel. (D) Spread platelets were also labeled with α-cortactin (top panel) and rhodamine phalloidin (middle panel). Merged images are shown in the bottom panel. Protein extracts from WT and HS1^-/- ^mice were blotted (E) for cortactin, WASp, Scar/WAVE1, Scar/WAVE 2 and N-WASp. Blots were also probed for tubulin to check loading. Scale bars = 5 μM.

As an F-actin and Arp2/3 binding protein, HS1 is predicted to play a role in the F-actin and Arp2/3 dependent processes of platelet shape change and spreading. To test this, the surface area of spread platelets was measured at 45 min after addition to immobilized agonists. Spreading of HS1^-/- ^platelets on CRP, collagen, fibrinogen or a combination of fibrinogen and thrombin was indistinguishable to that of wild type (WT) platelets both in terms of morphology and surface area (Figure 2A and2B). Spreading on fibrinogen alone was dominated by the presence of filopodia, whereas under all other conditions it was dominated by the presence of lamellipodia (Figure 2A &2C). In T-cells, it has been shown that the early signaling events downstream of the T-cell receptor that lead to formation of the immune synapse are intact in HS1^-/- ^mice, but that there is defective signaling to actin assembly and in maintenance of T-cell receptor signaling [17]. With this in mind, we monitored spreading at a later time point of 90 min, but again saw no change in platelet morphology on all surfaces relative to controls (not shown). In the case of platelets that had spread on fibrinogen alone, lamellipodia had begun to fill in the gaps between the filopodia and this accounts for the small increase in surface area relative to the 45 min time point; however, this was the same for both WT and HS1^-/- ^(data not presented). We have also observed platelet spreading in real time and again saw no difference between HS1^-/- ^and WT in the dynamics of spreading (not shown).

To further analyse spreading, HS1^-/- ^platelets were immunostained for the Arp2/3 complex and F-actin. HS1^-/- ^platelets displayed normal actin organization following spreading on fibrinogen, namely bright foci of F-actin and filopodia, that was indistinguishable from wild type platelets (Figure 2C). The Arp2/3 complex in these platelets, as identified using an antibody to the 34 kDa subunit, was localized primarily to the actin foci and to the cytoplasm. When platelets were spread on fibrinogen in the presence of thrombin, cells from both genotypes contained actin-rich stress fibers and the Arp2/3 complex was localized predominantly to the peripheral edge of the lamella (Figure 2C) although some foci of Arp2/3 were also observed in the cytoplasm. Again, no difference was observed between the WT and HS1^-/- ^platelets. Spread platelets were also immunostained for cortactin (Figure 2D) to establish whether a lack of HS1 altered the distribution of cortactin. No differences were apparent between the two samples when spread on fibrinogen ± thrombin or collagen indicating that cortactin distribution is normal in these cells. Together these data indicate that, in platelets, the re-organization of F-actin and Arp2/3 complex which underpins cell spreading does not require the activity of HS1.

It is feasible that other activators of the Arp2/3 complex are able to compensate for the loss of HS1 in platelets. We probed western blots of platelet protein extracts for cortactin, WASp, Scar/WAVE1 and Scar\WAVE2 to determine if there was any up-regulation of these proteins. The expression of cortactin, Scar/WAVE2 and WASp was the same in both WT and HS1^-/- ^platelets indicating that there was no compensatory up-regulation of these proteins (Figure 2E). We did observe a small reduction in the level of Scar/WAVE1 in HS1^-/- ^platelets compared to WT. However, we do not feel that this would be likely to have any significant effect as previous studies have shown that platelets from Scar/WAVE1 null mice have a relatively mild phenotype specifically downstream of GPVI signaling [19] and in the current study, no difference in the response of WT and HS1^-/- ^platelets to CRP was observed. We have also demonstrated that there is no platelet defect in the Scar/WAVE1 heterozygote (unpublished data). Western blots were also probed for N-WASp. No band was observed on these blots indicating that N-WASp, if present in platelets, is expressed at low levels (below the detection limit of this antibody on western blot) and that a lack of a HS1^-/- ^phenotype is not due to an increase in N-WASp expression.

**Figure 3:**
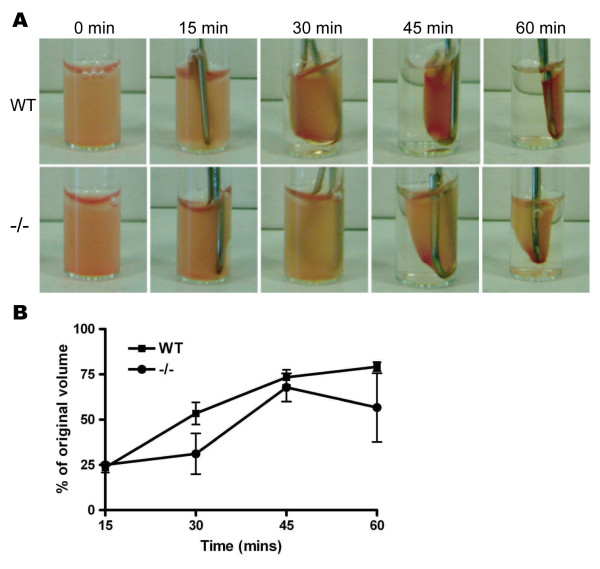
**Clot retraction assays in WT and HS1^-/- ^PRP**. (A) Time course of clot contraction and (B) the volume of serum excluded at each time point. No significant difference was observed between WT and HS1^-/- ^platelet rich plasma at any time point (n = 3).

The processes of clot retraction and thrombus stability which follow initial thrombus formation are known to be dependent on actin and myosin [2,20]. Accordingly we tested whether clot retraction was impaired in the absence of HS1 using platelet rich plasma from wild type and mutant mice. Images of representative clots taken at 15 min time intervals are shown in Figure 3A. The % of serum remaining in the tube after the clot was removed was measured for each time point. Whilst there appears to be a slight delay in clot retraction at 30 min, over the three replicates performed we observed no significant difference between the genotypes at any of the time points taken (Figure 3B). Nevertheless, clots formed in PRP from HS1^-/- ^mice were still able to retract to a similar level to that observed in WT PRP.

### Platelet aggregation and secretion

The number of platelets was measured following removal of whole blood from terminally-narcosed mice. WT mice had a mean platelet number of 496 ± 36 × 10^3^/mm^3 ^whilst HS1^-/- ^mice had a mean of 423 ± 36 × 10^3^/mm^3^. There was no significant difference (P = 0.124) between these two numbers demonstrating that HS1^-/- ^is not essential for platelet formation.

**Figure 4:**
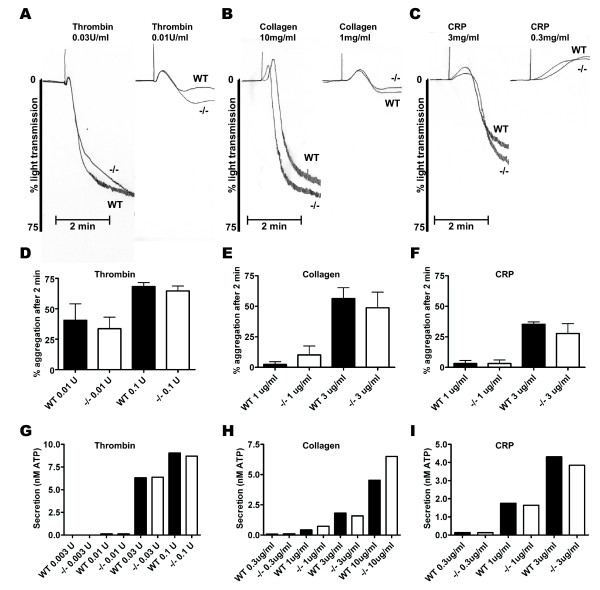
**Aggregation and secretion response of WT and HS1^-/- ^platelets**. (A) – (C) Representative aggregation traces for WT and HS1^-/- ^platelets in response to maximal and threshold concentrations of thrombin, collagen and CRP. (D – I) Percentage aggregation and ATP secretion of WT and HS1^-/- ^platelets was determined after 2 min stimulation with varying concentrations of thrombin (D & G), collagen (E & H) and CRP (F & I). For % aggregation the mean ± SEM of 3 different mice is presented. For ATP secretion, mean ± SEM from 1 representative experiment is shown (WT – Black bars, HS1^-/- ^– White bars). No significant difference was observed between WT and HS1^-/- ^platelets for any of the agonists tested.

In view of the fact that the actin assembly contributes to activation of PLCγ 2 downstream of the major platelet signaling receptor for collagen, the immunoglobulin GPVI [21], we investigated platelet aggregation and secretion responses to collagen and the GPVI-specific agonist CRP alongside responses to thrombin using WT and HS1^-/- ^platelets. Representative traces for aggregation to maximal and threshold concentrations of each of the three agonists are shown in Figure 4A, B, and 4C. Essentially, no significant difference in the pattern or extent of aggregation was observed between the two genotypes for all three agonists (Figure 4D, E &4F). Secretion of ATP from these platelets was monitored simultaneously with aggregation using luciferin luminescence. Again, no difference was observed in ATP secretion between WT and HS1^-/- ^platelets in response to the three agonists (Figure 4G, H &4I). This data, in conjunction with the spreading data presented above, indicates that abrogation of HS1 does not impair the ability of platelets to respond to stimuli and to successfully transduce the signals required for shape change, aggregation and secretion.

### Role of HS1 in thrombus formation *in vivo *and *in vitro*

**Figure 5:**
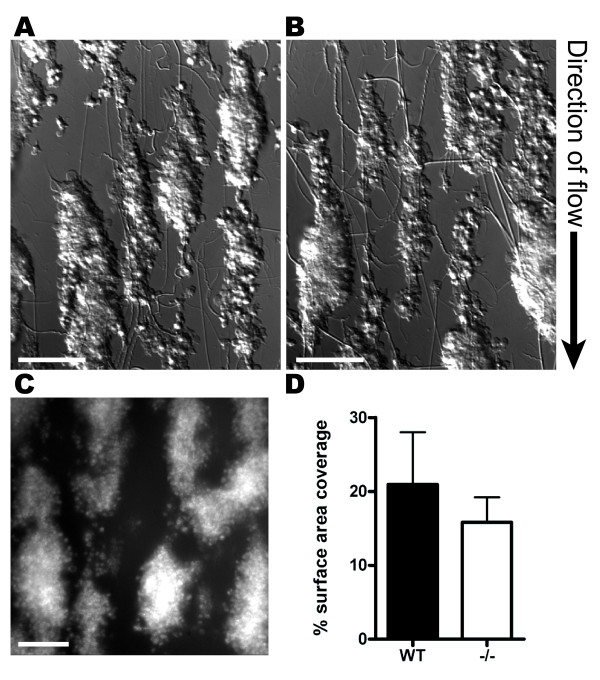
***In vitro *flow assays**. Whole blood from WT (A) or HS1^-/- ^(B) mice was flowed over collagen at a shear rate of 1000 s^-1 ^for 4 min. Aggregates were fixed in formalin and imaged using DIC. Flow experiments were also carried out with DiOC_6 _labeled platelets (representative image shown in C) and the mean surface area of aggregates was calculated from fluorescence images (D). Means ± SEM are plotted (n = 3). No significant difference was observed between surface area coverage of WT and HS1^-/- ^platelets (P = 0.669). Scale bars = 20 μM

We extended our studies to flow-based test systems to further investigate possible differences between WT and HS1^-/- ^platelets. The ability of platelets to adhere and form aggregates under high shear rates (1000s^-1^) was tested using *in vitro *flow experiments. No difference was observed between WT and HS1^-/- ^blood in the visual appearance of the aggregates formed during these experiments (Figure 5A &5B). Analysis of surface area coverage of platelets in 10 random field of view quantified using fluorescently labeled platelets (Figure 5C &5D) confirmed that there was no significant difference between the genotypes (P = 0.669, ns).

**Figure 6:**
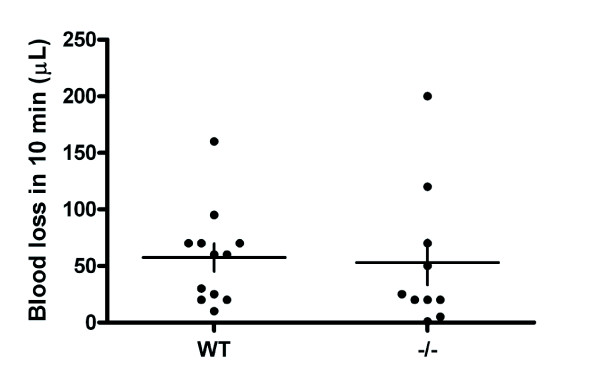
**Bleeding time measurements in WT and HS1^-/- ^mice**. HS1^-/- ^mice show no significant difference in bleeding as determined by volume of blood lost in 10 min following removal of the terminal 3 mm of the tail (P = 0.852). Closed circles represent individual data points, horizontal bars the mean and vertical bars the SEM. (n = 12 for WT and 10 for HS1^-/-^).

We also tested the bleeding time using a tail bleeding assays on HS1^-/- ^(n = 10) and WT (n = 12) mice. For these studies, the tail was laid flat and the volume of blood that dripped from the tail following removal of a 3 mm portion was recorded. The data collected was expressed as volume of blood lost in 10 min (Figure 6). No significant difference was observed between the two genotypes in the volume of blood lost in 10 min (P = 0.852, ns) demonstrating that absence of HS1 does not result in a bleeding disorder.

After this work was submitted, Kahner *et al *[22], published a paper which described tyrosine phosphorylation of HS1 in human platelets downstream of GPVI. This manuscript also described a mild bleeding phenotype for the HS1^-/- ^mice using tail bleed and FeCl_3 _*in vivo *injury models. Further, this study also reported a small increase in the time taken for platelets to change shape in response to convulxin and PAR-4 agonists although aggregation appeared normal. They also observed reduced dense granule secretion in null mice. It is feasible that these relatively small differences could be due to HS1's role in actin dynamics, although the authors do not discuss their results in relation to the actin cytoskeleton and indeed do not propose a mechanism for this defect. There is no clear explanation for the differences observed in the present study, although it should be emphasized that the relatively mild nature of the phenotype described by Kahner *et al *[22] emphasizes that the role of HS1, if any, is relatively mild. It is possible that differences between the two studies could be related to subtle differences in the genetic composition of the mice due to in-breeding (and therefore the presence of modifier genes), although it should be noted that both studies were performed on C57BL/6 background and the results were compared to those obtained on littermate controls.

The absence of a phenotype for HS1^-/- ^platelets is surprising in light of the work by Kahner *et al *[22], bearing in mind that it has a relatively limited tissue expression profile [23] and that it undergoes tyrosine phosphorylation in activated platelets [14]. It is possible that cortactin and HS1 could be functionally redundant as they share a very similar domain structure and that cortactin is highly expressed in megakaryocytes and platelets [24]. In contrast, B and T-cells, which show a distinct phenotype in the absence of HS1, express a low level of cortactin. It is therefore important to extend this work to platelets deficient in cortactin and in both cortactin and HS1.

## Methods

### Mice

HS1^-/- ^mice were a kind gift from Drs Takeshi Watanabe and Diasuke Kitamura (Kumamoto University, Japan). Mice were back-crossed into the C57BL/6 background and bred as heterozygotes. All experiments were performed on mice aged 6 – 10 weeks of age using litter-matched controls (designated WT). Observation of the mice revealed no obvious defects in development and HS1^-/- ^mice were visually undistinguishable from WT or heterozygote mice. Genotyping of mice was carried out by PCR on genomic DNA extracted from ear clippings taken at 3 weeks after birth. Primers HS1-3'KO-S (5'-GAGAGGAAAGGTAGACACCAG-3') and HS1-KO-end-3' (5'-GGCATGGATGGCTGCTGGAC-3') were used to identify wild type mice. HS1^-/- ^mice were identified using primers HS1-3'KO-S and reverse primer Lac-Z-3' (5'-CATGCTTGGAACAACGAGCGC-3'). All animals were maintained using housing and husbandry in accordance with local and national legal regulations.

### Preparation of murine platelets

Blood was drawn from CO_2 _terminally-narcosed mice under anesthetic from the hepatic portal vein and taken into ACD at a ratio of 1:10 or, for aggregation studies performed in platelet rich plasma (PRP), into sodium citrate. Platelet numbers in whole blood were determined using an ABX Micros 60 (ABX Diagnostics, Montpelier, France). PRP and washed platelets were prepared as previously described [19].

### DIC and fluorescence microscopy of spread platelets

Cover slips were incubated with a suspension of fibrinogen (100 μg mL^-1^), collagen (100 μg mL^-1^) or collagen related peptide (CRP, 100 μg mL^-1^) overnight at 4°C. Surfaces were washed and then blocked with denatured BSA (5 mg mL^-1^) for 1 h at room temperature followed by subsequent washing with PBS before use in spreading assays. Platelets (2 × 10^7 ^mL^-1^) were layered on immobilized proteins and allowed to adhere for 45 or 90 min at 37°C. Surfaces were then washed with PBS to remove non-adherent cells before fixation with 10% formalin, neutral buffered, for 10 min at room temperature. Platelet morphology was imaged as previously described [19]. The platelet surface area of spread platelets was computed using a java plugin for the Image J software package as previously described [25].

Immunolocalization of F-actin, Arp2/3 complex and cortactin was carried out as follows. Fixed platelets were made permeable in 0.1% Triton X-100 in PBS for 5 min, washed 3× in PBS and then incubated in α-p34 or α-cortactin for 60 min at room temperature (1 in 500 dilution in PBS). Coverslips were washed 3× in PBS and then incubated for 30 min with goat α-rabbit-488 or goat α-mouse-fitc and rhodamine phalloidin (1 in 500 and 1 in 1000, respectively). Samples were washed and mounted in Mowiol and imaged using a Zeiss 63× oil immersion Plan-Apochromat lens on a Zeiss Axioskop2 microscope. Digital images were captured by a Qicam Fast digital camera (Qimaging corporation) using Openlab 4.0.3 software (Improvision).

### Clot retraction assays

Whole murine blood was anti-coagulated with sodium citrate and PRP prepared as above. The platelet count was adjusted to 3 × 10^8^/ml with HEPES-Tyrodes containing CaCl_2 _(2 mM) and fibrinogen (2 mg/ml). 400 μl of this mix was placed into an aggregometer tube and incubated at 37°C for 5 min. 2 μl of mouse erythrocytes were added for colour contrast. Thrombin (10 U/ml) was added and mixed with a paper-clip and clot retraction was allowed to proceed at 37°C for 1 hour with the paper-clip present. At appropriate time points photographic images of retracting clots were recorded and the clot was pulled out with the paper-clip and the remaining serum volume measured. These experiments were performed blind.

### Platelet aggregation studies

Platelet aggregation was monitored using 300 μL of 2 × 10^8 ^mL^-1 ^of either PRP (for CRP and collagen) or washed platelets (for thrombin). Stimulation of platelets was performed in a Chrono-Log aggregometer (Chrono-Log, Havertown, PA, USA) with continuous stirring at 1200 rpm at 37°C as previously described [21].

### *In Vitro *Flow studies

For *in vitro *flow studies, mouse blood was prepared and treated as described by Calaminus *et al. *[19]. Platelet adhesion results are expressed as the percentage of surface area covered by platelets.

### Tail bleed assays

Experiments were conducted on 20–35 g male and female WT (n = 12), and HS1^-/- ^(n = 10) mice. Mice were anaesthetized with isofluorane via a face mask throughout the experiment and subsequently injected with the analgesic buprenorphine (ip). The animal was laid flat on a box of height 15 cm and the tail was laid horizontally along the box with the tip (1 cm) protruding horizontally over the edge. The terminal 3 mm of tail was removed using a sharp razor blade and blood was collected in a graduated 3 ml blood tube containing 1.5 ml H_2_O. Mice were allowed to bleed until they lost either 15% blood volume (which was calculated prior to the experiment based on the animal weight and assuming a blood volume of 70 ml/kg) or for 20 min. Data were presented as the volume (μl) of blood lost in 10 min.

### Data Analysis

Results are shown as mean ± SEM from at least 3 experiments unless otherwise stated. Statistical comparisons were made using Student's test or a non-parametric test.

## Authors' contributions

SGT designed and carried out the experiments and wrote the manuscript. SDJC assisted with carrying out the experiments. JMA performed the tail bleed and *in vitro *flow experiments. SPW and LMM conceived the study, assisted in designing the experiments and in writing the manuscript. All authors read and approved the final manuscript.
